# Perceived enablers and constraints of motivation to conduct undergraduate research in a Faculty of Medicine and Health Sciences: What role does choice play?

**DOI:** 10.1371/journal.pone.0212873

**Published:** 2019-03-13

**Authors:** Debra Leigh Marais, Jessica Kotlowitz, Bart Willems, Nicola W. Barsdorf, Susan van Schalkwyk

**Affiliations:** 1 Undergraduate Research Office, Faculty of Medicine and Health Sciences, Stellenbosch University, Cape Town, South Africa; 2 Faculty of Medicine and Health Sciences, Stellenbosch University, Cape Town, South Africa; 3 Division of Health Systems and Public Health, Faculty of Medicine and Health Sciences, Stellenbosch University, Cape Town, South Africa; 4 Health Research Ethics Office, Faculty of Medicine and Health Sciences, Stellenbosch University, Cape Town, South Africa; 5 Centre for Health Professions Education, Faculty of Medicine and Health Sciences, Stellenbosch University, Cape Town, South Africa; University of Newcastle, AUSTRALIA

## Abstract

**Background:**

Enhancing evidence-based practice and improving locally driven research begins with fostering the research skills of undergraduate students in the medical and health sciences. Research as a core component of undergraduate curricula can be facilitated or constrained by various programmatic and institutional factors, including that of choice. Self-Determination Theory (SDT) provides a framework for understanding the influence of choice on student motivation to engage in research.

**Aim:**

This study aimed to document the enablers and constraints of undergraduate research at a South African Faculty of Medicine and Health Sciences (FMHS) and to explore how the presence or absence of choice influenced students’ engagement with research in this context.

**Methods:**

An exploratory descriptive design was adopted. Undergraduate students who had conducted research and undergraduate programme staff were recruited through purposive sampling. Semi-structured interviews were transcribed and thematically analysed. Findings were interpreted using SDT, focusing on how choice at various levels affects motivation and influences research experiences.

**Results:**

Many of the programmatic and institutional enablers and constraints–such as time and supervisory availability–were consistent with those previously identified in the literature, regardless of whether research was compulsory or elective. Choice itself seemed to operate as both an enabler and a constraint, highlighting the complexity of choice as an influence on student motivation. SDT provided insight into how programmatic and institutional factors–and in particular choice–supported or suppressed students’ needs for autonomy, competence, and relatedness, thereby influencing their motivation to engage in research.

**Conclusion:**

While programmatic and institutional factors may enable or constrain undergraduate research, individual-level factors such as the influence of choice on students’ motivation play a critical role. The implication for curriculum development is that research engagement might be enhanced if levels of choice are structured into the curriculum such that students’ needs for autonomy, competence, and relatedness are met.

## Introduction

Enhancing evidence-based practice and improving locally driven research begins with fostering the research skills of undergraduate students in the medical and health sciences [1–4]. Exposure to research from an early stage in students’ careers is more likely to lead to postgraduate study [5,6] and involvement in research during their careers [6–12], thereby fostering a cadre of researchers and research-literate clinicians able to critically evaluate research findings for application in clinical practice [13–15]. There is, however, much variation with respect to the extent to which research is incorporated as a mandatory or elective component of undergraduate programmes and this remains a matter of some debate [10,16–19].

Of particular relevance for this study is the challenge of fostering student engagement in research, whether as an elective or a compulsory activity. Some have argued that making undergraduate research compulsory risks forcing students to participate in an activity in which they have no interest [19]; others suggest that exposing all students to research creates the possibility of sparking students’ interest in an activity (and subsequent career path) that they might otherwise not have considered [20]. The element of student choice thus adds an important dimension for exploration within the context of other programmatic and institutional enablers and constraints of undergraduate research.

### Programmatic and institutional enablers and constraints of undergraduate research

Incorporating research into undergraduate curricula in medical and health sciences faculties can pose several challenges. These include limitations on time and space in the curriculum [19,21–24], insufficient availability of infrastructural and supervisory support [1,12,21,24–29], and lack of exposure to and awareness of research opportunities [9,23,25,30], leading to negative or inaccurate perceptions about research [30–32] and perceived lack of competence and self-efficacy [12,25,28,31]. In the health sciences, the majority of research conducted is health research, involving human participants and requiring ethical approval. This presents another barrier in terms of navigating the bureacratic process and the time taken to acquire this permission in a time-limited curriculum [6,9,25,31,33].

Enablers of undergraduate research mirror its constraints. These include curriculum strategies such as teaching on research methodology, early exposure to and preparedness for research experiences, and elective research activities [6,10,34]; sufficient resources and formal infrastructural support [10,23,27,33–35]; bolstering supervision capacity and support [9,33,35,36]; doing research in groups [6,12,37]; and creating and raising awareness about research opportunities and fostering a sense of belonging in a research environment by acknowledging students’ research achievements [9,10,23,24,34,35]. In addition, there are individual-level factors (such as personality, motivation etc.) that need to be taken into account [25,38,39].

Previously, undergraduate medical students have reported the need for inclusion of research in the core curriculum to facilitate the process [21,32]; however, many staff and students still believe that research should be an elective for medical and health sciences students [22]. In South Africa, where this work was undertaken, the Health Professions Council of South Africa (HPCSA) requires all allied health students (Human Nutrition, Occupational Therapy, Physiotherapy, Speech-Language and Hearing Therapy) to complete research projects as a compulsory component of their degrees, while for undergraduate medical students, research is optional. Nonetheless, some universities have integrated research as part of the core curriculum for medical students [4,27], as engagement in research, particularly self-directed research, is seen as complementary to an evidence-based health care approach [36,40], as well as fostering a critical skill set in relation to the HPCSA’s core competencies of health professionals [41].

### Influence of choice on motivation and engagement: Self-Determination Theory

At the centre of any learning and teaching activity is the individual student. It is therefore important to consider their motivation for participating in research activities. Giving students choice over tasks required of them can affect students’ intrinsic motivation to engage with the activity [42–45]. Self-Determination Theory (SDT) [46,47] is a theory of motivation that can help understand how or why this happens [42,44]. It suggests that our need for autonomy, competence, and relatedness must be satisfied in order for us to be motivated [46,47]. SDT defines autonomy as self-initiation and self-regulation of one’s actions [46]. Competence involves understanding how to achieve various external and internal outcomes and being efficacious in performing the requisite actions [46]. Relatedness speaks to the development of secure and satisfying connections with others [46]. Social contexts–including learning contexts–that satisfy these needs could enhance intrinsic motivation [46–48] which has been linked to various advantageous educational outcomes [46,49].

Deci and Ryan [46] suggest that motivation will be enhanced where support for competence and relatedness are in place but that intrinsic motivation will only be enhanced if these competence- and relatedness-supportive elements are also autonomy-supportive. There seems to have been particular emphasis given to autonomy in SDT studies [42], with arguments that autonomy is a necessary, but not sufficient, condition for student motivation [44,46]. In principle, then, it is easy to assume that providing choice is always beneficial and should increase motivation. However, this does not always seem to be the case. Findings that show the positive effects of choice on motivation [44,46,50] have been mediated by others showing that choice can either have no effect or even a negative effect on motivation [43,51]. Giving students more autonomy by providing more choices may increase their cognitive workload [52] and therefore negatively affect their sense of competence. This suggests that choice is a multifaceted phenomenon and that the association between choice and motivation is complex and variable [42].

SDT is a useful theoretical lens for the purposes of our study because it recognises that context affects motivation through influencing the extent to which these needs are met. Motivation is not a stable, dispositional trait but rather a situated and domain specific variable [49] and can be maximised depending on the extent to which the social context provides students with the opportunity to satisfy their basic psychological needs for autonomy, competence, and relatedness [46,53,54]. Contextual factors that have been identified as autonomy, competence, and relatedness supportive are listed in Table 1 below.

**Table 1:** Contextual factors that are supportive of autonomy, competence and relatedness needs.

Autonomy-supportive	Provision of choice with minimum pressure[6,46,48,55,56] Optimising sense of individual control [46,57] Encouraging self-initiation [48,56,58,59] Being able to realise personal goals, values, and interests [48,56] Clarifying the relevance of expected behaviours [48,55,60]
Competence-supportive	Structure [6,48,55,61] Understanding how to achieve various external and internal outcomes [46] Feeling/being efficacious in performing the requisite actions [46] Positive feedback about performance [46,55,56,58] Providing optimal challenges [46,55–57] Understanding the perceived utility/relevance of the activity [6,46]
Relatedness-supportive	Developing secure and satisfying connections with others [46] Interpersonal involvement of educators [46,48,62,63] Peer acceptance [46] Feelings and perspectives acknowledged [46,58] Opportunities to collaborate with others [6,57,63]

SDT as a framework allows us to explore how we might change contextual-level factors, including individual choice, in order to stimulate motivation and increase engagement (with research) and therefore improve student performance, as well as students’ perceived experiences of research. This study therefore responds to calls for research to better understand the contexts in which students’ motivated beliefs and behaviours occur [64] and, more specifically, to maximise the beneficial effects of choice as a contextual influence on motivation for individual students within a given curriculum [65]. It aimed to explore how to optimise students’ experiences of research by exploring common enablers and constraints of undergraduate research. A secondary aim was to understand whether giving students the choice to do research might facilitate positive experiences of research by enhancing motivation and engagement.

## Methods

### Study setting

This study was conducted at a Faculty of Medicine and Health Sciences (FMHS) at a South African university that places research at the core of its strategic objectives. The focus was on five undergraduate programmes at the FMHS in which research is conducted: Human Nutrition, Occupational Therapy, Physiotherapy, and Speech-Language and Hearing Therapy (the allied health programmes), where research is a compulsory component of these four-year degrees, and the MB,ChB (medicine) programme, where research is an elective, typically undertaken in the final (6^th^) year as individual research projects which can generate additional credits. Annually, about 130 students (i.e. all 4^th^ years) conduct research, usually in groups, across the four allied health disciplines; between 5 and 15 MB,ChB students (out of a 6^th^ year class of roughly 300) elect to do research. To get a complete picture of the enablers and constraints of undergraduate research in the faculty, we aimed to recruit students from both types of programmes (compulsory and elective).

While research is compulsory in the allied health programmes, students have a degree of choice with regards to their research topics, supervisors, and group members. Apart from Physiotherapy, where students do systematic reviews, these students conduct primary research, across a variety of qualitative and quantitative methodologies. A sample of project titles can be found in S1 Table. In the MB,ChB programme, students are exposed to teaching on evidence-based health care and can elect to use 4-week elective blocks in 4^th^ and 5^th^ year to work towards a final year research project. In general, MB,ChB students determine their own topics and have to approach relevant faculty members to act as research supervisors.

At the time of data collection for this study, the Undergraduate Research Office was being established to provide support to all undergraduate students conducting research in response to previous work undertaken at the FMHS which documented students’ understandings and perceptions of research [66]. This study builds on that earlier work.

### Study design

This is an exploratory descriptive study, located in an interpretive paradigm. The paper describes the perspectives of undergraduate students who conduct research and the staff who supervise them towards answering the research question, *what factors currently enable or constrain undergraduate research at the Faculty of Medicine and Health Sciences*?

### Participation selection

Participants were recruited using purposive, snowballing sampling. All students registered for full time undergraduate degree programmes in the FMHS who were conducting or had conducted research as either a core or extracurricular degree component were included. Full time staff members who had either been involved in supervising or coordinating undergraduate student research were also invited to participate.

Response rate was poor, despite repeated efforts to recruit participants from the relevant staff and student groups. Our final sample consisted of ten students and eleven staff members (N = 21, see Table 2) who indicated their willingness to participate, at which stage we ceased further recruitment as no new themes were being found during analysis. All staff and students interviewed from the allied health programmes were female. All MB,ChB staff participants were male; 1 of the four MB,ChB student participants was male.

**Table 2:** Participant details.

	Allied Health programmes				MB,ChB programme	Total
**Division**	Human Nutrition	Occupational Therapy	Physiotherapy	Speech-Language & Hearing Therapy	Various	
**Students** ^a^	4	2	0	1	4	**11**
**Staff** ^b^	0	2	2	2	4	**10**
**Total**	**4**	**4**	**2**	**3**	**8**	**21**

^a^ Student participants designated as S1, S2 etc. in interview excerpts below

^b^ Staff participants designated as ST1, ST2 etc. in interview excerpts below

### Data collection

Potential participants were contacted by email. Those individuals who consented to participate were interviewed for approximately 45 minutes to an hour, either face-to-face or over the phone, at a time convenient to them. With participants’ consent, interviews were audio-recorded for verbatim transcription following the interview. Interviews were all conducted by the same researcher (JK) using a semi-structured interview schedule (S1 Appendix) that was developed based on a comprehensive review of the literature on enablers and constraints of undergraduate research. At the time of this study, JK was a postgraduate student in the FMHS who was employed as a temporary research assistant and trained to conduct qualitative interviews. JK was therefore considered the best-placed member of the team to conduct the interviews, as all other team members were staff in the Faculty, which may have influenced student and staff participant responses.

### Data analysis

Data was coded using NVivo 10 and analysed using thematic analysis [67] to identify key themes.

The transcription of the data is itself believed to be a phase of data analysis [67]; having transcribed (verbatim) the data, JK already had thorough knowledge of the data set. Once transcription was completed, two researchers (JK and DM) engaged in repeated readings of the transcripts (initial reading through of all transcripts and then beginning to identify patterns and themes) to begin to code for key themes deductively, guided by codes identified after extensive engagement with the literature. From the initial list of ideas, JK and DM met to develop an initial coding framework. This framework was circulated to all team members with a sample of interview transcripts for review, after which the team met to discuss and refine the coding framework based on each member’s input regarding the need for additional or redundant codes. At this stage, we began to organise codes into broader categories or themes.

The refined coding framework (S2 Appendix) was then used by JK to code the entire data set in NVivo and organise the coded data into the broader themed categories. DM and JK then reviewed the results, refining identified themes to enable the full team to “organise the analysed data into a coherent and internally consistent account, with accompanying narrative … ensuring that each theme coheres around a central idea or concept” [67].

Notwithstanding the limitations regarding participant recruitment, our goal was to establish theoretical saturation [67,68] whereby data was collected to “exemplify theory … rather than to develop or refine theory” [69]. Thus, the extent of data collection and analysis is determined by the theory–that is, “given the theory, do we have sufficient data to demonstrate it?” [69]. While it is acknowledged that saturation is a matter of degree [70] and that there is always the potential for something new to emerge [69], saturation was determined to be reached in this study at the point at which further data collection would have been counterproductive [71,72].

Frambach et al.’s [73] criteria for ensuring quality (trustworthiness) in qualitative were used as a guideline for establishing the credibility, dependability, transferability, and confirmability of this study. Prolonged engagement and familiarity with the data [74] and investigator triangulation [75] were used as key strategies for ensuring the credibility of the data. Team members met regularly to review transcripts and revise the coding framework and to check the consistency and applicability of codes across the data set. Records were kept of the iterative data collection and analysis steps [73,76], as described above, in an effort to ensure dependability of findings. Verbatim extracts are also included to enhance the dependability of interpretations made [77,78]. Transferability is established by describing in detail the context of this study, noting that the goal is to achieve analytical, not statistical representativeness [79]. The role of the interview has been clearly explained in order to limit the threat of reactivity and therefore confirmability of the findings [80]. The validation of interpretations by multiple team members also served to limit researcher bias. Ethics approval was obtained from the Health Research Ethics Committee at Stellenbosch University (N14/03/026).

## Findings

The findings presented here relate to key factors that emerged as enablers and constraints of undergraduate research in the FMHS. Because choice emerged as one of these key factors, the focus moves to consideration of how choice itself was perceived as an enabling or constraining factor at different levels within the different programmes.

### Programmatic and institutional factors

There were many commonalities in terms of the programmatic and institutional perceived enablers and constraints across compulsory research and elective research programmes. Enablers included providing structured time and space within the curriculum to conduct research; exposing students to research early on in their programmes, and making clear links between theoretical concepts and practical applications; creating a research culture with research-related awareness and sufficient opportunities for students to become involved in research; facilitating ethics and other institutional permissions by providing adequate information and support; and providing varying degrees and combinations of choice around several aspects of the research process. Constraints were largely found to be the inverse of enablers: where sufficient time was found to be an enabler, for example, the lack of sufficient time was perceived to be a constraint, and so on.

#### Structured time and space in curriculum

Overcrowded curricula and insufficient time were challenges faced in both compulsory research and elective research programmes. Time was a limiting factor not just in terms of doing research but also in getting the necessary permissions to conduct research, as one student observed,

“There’s not much more they (the ethics committee) can do. It’s a long process that they have to do…It’s more on the students’ side, where we have to make provisions for the time that it’s going to take” (S3).

It was also perceived by staff to be a challenge in terms of the significant time investment required of supervisors:

“It’s a lot of time that you put in and sometimes you can’t publish the final product” (ST2).

For compulsory students, working in groups was perceived to be a facilitative factor in countering time constraints. When asked how their experience might have been different if working alone, for example, one student exclaimed,

“Oh, I would’ve died! Just because of the workload. The workload is crazy” (S8).

#### Early exposure linking theory and practice

Many suggested that starting research early in the degree programme was an enabling factor that might mitigate these constraints. However, this early exposure was perceived to only be effective if theory was linked to practical aspects of research in real time–that is, with outcomes that students need to produce as part of their research projects in order to consolidate theoretical concepts:

“Yes, they do theoretically prepare us for research but I don’t think anything can really truly prepare you for research until you’ve actually done it yourself and you realise that wow, okay, this is what it’s like” (S11).

This link is not currently being made sufficiently. Unsurprisingly, elective research students in particular felt underprepared to undertake research, given the lack of structured content on doing research in the curriculum.

“Students are taught about research in a very clinical trial-oriented manner, which is far, far above anything a student would ever try to attempt” (S7).

The absence of soft skills development of the more practical aspects of research–such as academic writing and referencing as well as setting up timelines and getting funding–was also identified as a constraint across programmes.

“Each time it kind of rehashed the same mathematical concepts but never really taking it through, this is how you develop a protocol, this is how you apply for ethics at this university, this practically how you access information…” (S7).

#### Creating an enabling environment

Poor awareness regarding what resources were available in the Faculty in aid of undergraduate research was identified as a constraining factor. Creating a research culture within the Faculty could serve to promote research and facilitate greater awareness of both opportunities and resources. The Faculty’s Annual Academic Day–a potentially ‘safe space’ at which students could present their work–was seen as an important mechanism in this regard.

“We’ve got an obligation to give them that platform to present, otherwise why are we doing this?” (ST10).

Challenges with respect to presenting and publishing undergraduate research were linked to time investment after graduation for both students and staff, a lack of funding, and a lack of sufficient opportunities, particularly in the allied health sciences.

“I don’t think undergrad students should be burdened with the responsibility of funds, being pressurised into producing publishable research, because it defeats the purpose of the exercise…it’s a learning exercise” (ST7).

Students were less likely than staff to identify lack of funding as a barrier.

“If there’s no funding for a project, then obviously it won’t happen” (ST9).

Staff highlighted the importance of funding particularly with respect to publishing or presenting student research, with many programmes exploring creative solutions to limited funding such as slotting student research into larger staff (funded) projects.

“So, we used funding from our department from a project that was left, you know, funding that was left over from a project and if it wasn’t for that I mean the students wouldn’t have been able to do the quality research that was in the end possible” (ST3).

#### Facilitating institutional permissions

The process of obtaining ethics approval was generally perceived as a significant barrier by students across both compulsory and elective programmes. Although there was recognition that this was a necessary part of the research process, many felt that the administrative requirements were unnecessarily burdensome for undergraduate students.

“I really felt that the current system for getting institutional permission…is so clumsy and disorganised” (S5).

Perceptions differed with respect to how this and other permissions processes could be facilitated, with some placing responsibility on the students, some on supervisors and some on the Faculty.

“We must go out of our way, not to bend rules, but to make it as easy as possible for them to do it…any delays will significantly deter students from doing research” (ST10).

Establishing a dedicated committee for undergraduate research was thought to be an important means of addressing these challenges, as was making information about required permissions easily available.

“I think the sort of support I wanted was making the muddle of administration easier and clearer” (S5).

### Individual choice: Enabler or constraint?

The empirical distinction between the two kinds of programmes along the lines of choice in this study allowed us to further unpack enablers and constraints of research at the level of the individual. This revealed some unexpected commonalities and differences in the experience of research–and associated enablers and constraints. Choice itself seemed to operate as an enabler or a constraint, regardless of the presence or absence of programmatic and institutional factors. These findings are discussed in relation to four different kinds of choices: choices about research participation, choice about research topics, choice about research supervisors, and choice about research groups.

#### Choices about research participation

Many MB,ChB students perceived the elective nature of research to be a disadvantage of the programme because their own experiences had exposed them to the intrinsic and extrinsic benefits of doing research, which they believed would be of value for all students:

“I really love research…I realise that lots of my classmates don’t necessarily like research, but I still think it’s important for them to learn about it. And I think the best way to learn about research is just to actually do it” (S5).

The current framing of the elective research project meant that it was perceived to only be an option for high achieving students. Paradoxically, then, choice seemed to operate as a mechanism of exclusion, limiting research to those who perceived themselves to be sufficiently competent to engage in it which, in turn, might have negatively affected the majority of students’ motivation to do so. As one student commented:

“It aims the research opportunity more towards your students who are aiming for distinction, which is a small portion of your class. And I think that’s a bit unfortunate. I think that makes research a pie in the sky for most students who say, ‘No, it’s not for me. It’s for the students that want to do very well’” (S7).

Conversely, MB,ChB programme staff tended to view the potential of *removing* choice in more negative terms, suggesting that students who are interested in research would benefit most from doing it and that those who are not should not be required to do so:

“You cannot force a student who’s absolutely not interested in research to do a full-scale research project, is my feeling” (ST1).

These participants thus seemed to be emphasising autonomy-related component of motivation, perhaps at the expense of the needs for competence and relatedness included in SDT. This could also highlight a perception, held by some MB,ChB staff, that clinical work and research work are somewhat separate endeavours:

“We’re not trying to train researchers, we’re trying to train clinicians and from those cherry pick those that could follow a career in research” (ST8),

whereas allied health staff tended to emphasise the intrinsic benefits of research for enhancing good clinical (evidence-based) practice:

“It’s first of all a professional pre-requisite of the HPCSA and of our profession that you need to be able to engage with research and also conduct research to add to the body of research in your field…And then secondly it teaches them to, I think, to become aware of what is available, of the wealth of information that is already available in the field and in the profession” (ST3).

“The doctors, they don’t understand research methodology. Which is really sad actually, because it really changes the way you practice as well” (ST7).

For students in the allied health programmes, the lack of choice was not necessarily perceived as negative; in fact, some students who may not have otherwise chosen to do research recognised the benefits in terms of enhancing their confidence and competence now that they had been exposed to it:

“I used to be very wary about [research] and quite nervous about it, but now that I’ve done it, I feel like I’ve been prepared to do that if I have to do it by myself” (S3).

This did not, however, lead to increased interest in or motivation to pursue further research, which was captured by one student as:

“No, I was never particularly interested in research and didn’t *not* enjoy doing research, but it certainly didn’t spark a huge interest. So I’m unchanged” (S10).

In contrast, the MB,ChB students unanimously expressed a desire to engage in research in the future:

“…because I know from the previous study how much you actually learn from it and how much you personally benefit, but also what power it has to bring about change” (S5).

However, this may be attributed to the fact that these students opted to do research in the first place and therefore were already interested in these pursuits.

Along with the choice to do research in the MB,ChB programme are associated choices regarding the nature of research (e.g. primary or secondary), topic, and supervisor. Although students in the allied health programmes have no choice about doing research, they are presented with varying degrees of choice further down the line–around topics, supervisors and group members. Again, choice was not experienced in exclusively positive or exclusively negative terms at these levels: there were ways in which it seemed to operate as an enabler and ways in which it operated as a constraint. While this was partly influenced by programmatic or institutional factors, it also demonstrated that choice is experienced in different ways by different individuals.

#### Choices about research topics

Allowing students to choose their own topics and thereby take ownership of the research project was seen by many staff as a mediating factor against negative experiences of research:

“Sometimes the students are not so invested in research as they would be if they could choose their own topic. So at the moment it’s anything goes and the role of the supervisor is just to make it into a do-able, meaningful project” (ST3).

Nonetheless, while there seemed to be greater potential for choice around topics than around the research component itself for allied health students, staff seemed to try to direct students towards particular research areas or encourage students to choose pre-determined topics:

“We will have all of the staff talking to the students and telling them what their area of interest is and if there are any research projects that they have going on where students can slot in to. And then students based on their interest are matched with supervisors” (ST5).

“What we do is we try and give them guidance in terms of topics because what we’ve also learnt over the years you know, is it’s quite labour intensive for us…and it gives them clearer direction as well” (ST6).

As a result, allied health students spoke primarily of having been assigned topics. In all cases, however, students perceived this lack of choice to be more of an advantage than disadvantage. There were several ways in which the absence of topic choice was enabling in these cases. In part, this was influenced by supervisory capacity constraints, which was offset by incorporating student research into existing research projects or aligning them with departmental research objectives, as well as by the increased potential of publishing such research.

Some students also reported that they felt overwhelmed by having to choose their own topics and were glad that this choice was largely directed for them:

“I think if we had to choose, it would have been such a difficult decision to make…so if you are just put into one of the (topics), you just work hard and you go, ‘we need to do it’” (S3).

Students recognised that staff were more in touch with key research areas or relevant questions and were thus happy to be guided by staff in terms of research topics:

“If you’re doing a research project you might as well contribute to the profession. So now the lecturers give topics. Which I think is really good because they are constantly busy with the information that’s going on and they can see where the gaps are and they can think ahead about the research almost. So that’s a good thing” (S8).

Some students described becoming more interested in the topic as the research progressed:

“I think when you first read a topic you think, ag [no] …but the more you work with a topic, it becomes a passion” (S8).

while others suggested that having no choice in their topic did not affect their interest in continuing to do research:

“So the topic is not my main interest but ja, research-wise, I’m open for more research” (S6).

#### Choices about research supervisors

In most cases, where allied health sciences students were assigned topics, these were attached to staff members who would become their supervisors. The absence of choice regarding supervisor selection did not seem to be perceived in either overtly positive or negative terms by these students. Some allied health staff noted that students sometimes seemed to prioritise choosing the supervisor they wanted to work with over choosing their own topic. When commenting on topic choice, one staff member observed that:

“It happens the other way around, that they would come to you and say, you know, I really want you as a supervisor, give us your topic so we can push it…” (ST6).

In contrast, MB,ChB students valued being able to select their own supervisors, with many recognising the importance of a good working relationship, supportive feedback, and supervisor availability in enhancing their research experiences:

“Even if we did silly things, he was just so supportive and sort of told us we were doing a good job. It was a very positive experience with him…(He) was also really busy but somehow we could always make a plan to see him” (S9).

“We kind of had supervision the entire time which is lovely… I do think that, you know, it is a scary thing for students just to go about because there’s so many things that you have to take into consideration, so just having the support of a supervisor is essential” (S3).

The importance of the supervisory relationship was highlighted by several students:

“Choose a supervisor who you seem to get along well with or seem to admire…” (S7).

“I think approaching the right supervisor is key. Getting someone that would support you enough, that’s absolutely key” (S5).

While choice was generally regarded as an enabling factor for MB,ChB students, this could be seen as somewhat dependent on creating an enabling environment that could facilitate the choices that these students had:

“I think that apart from students taking their own initiative, there [should also be] researchers that specifically make time to talk to students about research opportunities” (ST9).

“I think, one would need to create a research culture and that research culture should be instilled in senior colleagues who would then need to mentor junior colleagues. … Students are motivated. Students are very, very talented students. It’s the senior colleagues who are not always interested in research and some of them feel that they don’t want to invest in students” (ST10).

The absence of structures or platforms in which students have access to staff projects or ideas that they could link up with might create a situation in which too much choice becomes overwhelming or obstructive. Mirroring the ways in which the allied health programmes had needed to accommodate large groups of students doing research (through assigning research topics, for example), staff in the MB,ChB programme recognised that making research compulsory–that is, taking away choice–would be a constraint on existing supervisory capacity:

“I think there’s a lot of opportunities. I think there’s no shortage of study questions and I think a lot of stuff can be done retrospectively… But I think the bigger challenge would be to actually get supervisors who are willing to take them through this process” (ST10).

Allowing MB,ChB students to choose their own supervisors similarly enabled staff to exercise the choice of whether or not to take on undergraduate student researchers and generally meant that they had the positive experience of working with enthusiastic students:

“It’s very rewarding to see somebody being involved in research that is very keen on doing it. … because it’s at the moment an optional thing for the students, those that do it are really very keen to do it. And it’s very rewarding to see that kind of optimism for a project” (ST8).

#### Choices about research groups

In all allied health programmes, students conducted research in groups, primarily due to pragmatic and logistical considerations regarding large class numbers. Staff highlighted several indirect benefits of this compulsory group work:

“…especially if it’s a group research project, ja, it doesn’t always go smoothly, so sometimes I think they pick up very important skills for conflict management and time management” (ST3).

However, these were also often identified as potential challenges of group work, as was the possibility that stronger students might carry the workload for weaker students:

“But I mean, with any research study you have those who take the lead and you have those who necessarily kind of fall back and let the others do the work” (S3).

Several strategies had been implemented to mitigate against this risk. In most cases, students were allowed to choose their own group members. This also had the potential to be an enabling or a constraining factor: conflict arose even where students had chosen their own groups:

“These are people that I selected to work with, I was not assigned to work with them. And still we found that some of us were doing the majority of the work” (S7),

while students who had been allocated to groups recognised the value of bringing together members with diverse backgrounds and skill sets:

“working with people you don’t normally work with is always a good thing because you learn so much from them as well” (S3).

Whether the choice of working in groups and the specific groups chosen was prescriptive or optional, all students reported that they would still choose to work in groups if they could do it again, identifying a number of benefits from working collaboratively:

“I would’ve struggled more [alone]. I like to have people to work with and bounce ideas off of and that sort of thing so I did enjoy working in a group” (S10).

“We learnt a lot from each other and we could bounce ideas off each other and when I kind of really hit a dip, someone would be on the ball. But mostly, I think I actually had the guts to do it because we were three and I wouldn’t have managed it by myself” (S9).

In contrast to allied health students who seemed to prefer working in groups, MB,ChB students, for whom it was mandatory to work alone, generally seemed prefer this:

“I think what was nice for me working on my own in this last study. I think it’s nice, I don’t know I’m quite a, I actually like working on my own. Just picking a field that you are very comfortable in, not having to sort of discuss as a group everything, you can just go ahead” (S5).

In this case, then, having no choice but to work on individual projects was perceived as enabling. Some of these students had had prior experience of working in research groups and had found the potential for conflict to be a constraining factor, reiterating the value of having to work independently. It seems that the one MB,ChB student who had only experienced doing research as part of a group (i.e. not as a final year project for assessment) had, like the allied health students interviewed, perceived this to be enabling rather than constraining:

“I would probably still work in a team. I like that… I think that I would’ve gotten very demotivated and certain things would’ve just taken me longer that aren’t my strengths, so especially the amount of time and it just not being as fun” (S9).

“…within our group, everyone motivated each other and those who don’t always work as hard, really, it was nice to see them getting excited and doing their work and trying their best. So I think that you always need that, you need people who will motivate each other to work hard” (S3).

This is a clear demonstration of how working with others was a motivating factor.

## Discussion

Previous research has shown that undergraduate research has several benefits for students [24,81–83]. Encouraging research at undergraduate level has the added advantage of developing a cadre of much needed clinician scientists as well as research-literate clinicians [6,8–12,84–86]. Findings of this study with respect to enablers and constraints of undergraduate research were found to be consistent with literature, regardless of whether research was a compulsory or elective component of the relevant programmes. The issue of choice, however, offers new insights concerning how choices around research participation, topics, and groups can enhance motivation though meeting students’ needs for autonomy, competence, and relatedness.

While it might be anticipated that giving students more choice/s would positively affect their experience of the activity at hand, the findings of this study suggest that choice is a complex construct that has a variable effect on motivation and engagement. Students who were not given a choice reported a positive experience of research and in some instances perceived the absence of choice to be an enabling factor. However, making research compulsory and structuring it into undergraduate programmes did not necessarily mediate what were perceived to be barriers, such as insufficient time and administrative burdens.

Similarly, students who chose to do research (MB,ChB programme) experienced challenges associated with having no specific content or time around research built into the curriculum. This lack of structure was also highlighted by MB,ChB programme staff, where research was elective. But the elective students also recognised the logistical and capacity-related implications of restructuring the MB,ChB programme to incorporate research, with potentially negative effects on students’ experiences of doing research. In this respect, optimising the opportunity to do research was seen as strongly dependent on students’ own initiative as well as on the initiative of staff and the faculty generally to create an enabling environment (research culture) to facilitate this.

In this discussion, we use Self-Determination Theory (SDT) relating to students’ needs for autonomy, competence, and relatedness as a theoretical lens to make sense of the findings discussed above. Specifically, SDT is used as an organising framework to understand how the nature of choice affected students’ motivation to engage in research. We draw on findings to highlight the autonomy-, competence- and relatedness-supportive contextual factors that may enhance student motivation.

### The need for autonomy

Autonomy has tended to be foregrounded as a necessary but not sufficient condition for motivation in much of the SDT research. In this study, autonomy played out in terms of the choice to participate in research or not. The absence of choice at this level, however, did not preclude opportunities for the exercise of autonomy further down the line. At these levels, it is interesting that complete autonomy or blanket choice (for example, in relation to topic choice) was sometimes experienced as incapacitating (i.e. by allied health sciences students), in part because students felt overwhelmed. This suggests that ‘satisfying’ the need for autonomy sometimes came at a cost to the need for competence. Interestingly, this also played out in the converse. Having autonomy to choose to participate in research or not–as was the case with the MB,ChB students–was perceived by these students as having a negative effect on students’ *perceived* competence to engage in a task that, by virtue of this choice, was seen as exclusive to only high achieving (i.e. competent) students.

The provision of choice seemed to be one of the most significant autonomy-supportive programmatic factors in this study. At the level of the choice to participate in research–i.e. for MB,ChB students–this then facilitated other autonomy-supportive elements identified in the literature, such as individual control, self-initiation, and realising one’s own goals, values, and interests [46,48,87]. This was also the case, although to a lesser degree, for allied health students who were provided with limited choices regarding their research topics, supervisors, and research group members. Consistent with SDT, these autonomy-supportive factors seemed to enhance motivation. As mentioned above, however, the *absence* of choice, although construed as autonomy-suppressive [46,48], was not necessarily a constraint on student motivation. In some cases, this actually served to provide students with sufficient structure to feel capable of engaging in research. This demonstrates the importance of ensuring that the other needs underlying students’ self-determination, such as the need for competence, are met. Clarifying the relevance of the activity has also been identified as an autonomy-supportive factor [48,55,60]. Many of the MB,ChB students in this study certainly seemed to understand the relevance of research to their clinical contexts and for health care in general and were strongly motivated by this. In contrast, although allied health staff emphasised that research is integral to good clinical practice, this did not come across as a factor affecting allied health students’ motivation or engagement with research.

### The need for competence

There was a strong sense that all the elective (MB,ChB) student participants felt high levels of competence and self-efficacy as they tended to be academically strong, high achieving students. This could explain their motivation to engage in research. Thus, it may not have been so much that the element of choice satisfied their need for competence, in this case, as it was that their (pre-existing) competence enabled them to make the choice to do research. However, these students did express frustration regarding lack of information and support regarding how to obtain the necessary permissions for their research, for example, which seemed to have an undermining effect on their sense of self-efficacy and competence. This is consistent with research which suggests that knowing how to achieve internal and external outcomes is a competence-supportive factor [46]. This is important to keep in mind when trying to enhance student motivation through optimising their need for competence, by having sufficient structural and administrative support in place and access to information regarding processes and requirements. Without this, the autonomy-enhancing aspects of choice, which for some students could have increased motivation, may be counteracted.

The lack of structure seemed to be experienced by MB,ChB students as competence-suppressive; these students persisted in spite of these frustrations, which appeared to be largely due to their individual characteristics and pre-existing motivation to engage in research. While allied health students seemed to feel less confident about research than their MB,ChB counterparts, the structure provided by their programmes was competence-supportive to the extent that the allied health students reported positive experiences and greater confidence at the end of the research process. Allied health students tended to be much less confident in their perceived competence to engage in research, despite the high degree of structure–a competence-supportive factor [6,48,55,61]–and support built into their programmes. Being able to meet this need seemed to depend to some extent on having limited degrees of autonomy (e.g. could choose research group but not research topic) in terms of having some but not complete control over research-related choices, as discussed above. This links to the importance of providing optimal but not excessive challenges in order for students’ need for competence to be realised [46,55,56]. In addition, competence was enhanced in allied health students’ case by sharing the load with others, which could arguably be experienced as somewhat restrictive of autonomy. In contrast, being able to realise their need for autonomy by working alone seemed to have the effect of enhancing MB,ChB students’ sense of competence.

Positive feedback about performance also appeared to boost students’ sense of competence, regardless of whether their research was compulsory or elective. Consistent with clarifying relevance as an autonomy-supportive behaviour, if students understood the perceived utility of the activity, their need for competence seemed more likely to be met [46].

### The need for relatedness

In this study, students’ need for relatedness could be met through relationships with supervisors and, for allied health students, through relationships with members of their research groups, enabling students to develop secure and satisfying connections with others [46]. Some students explicitly stated that working with others was motivating. The interpersonal involvement of educators through supervisory relationships has been identified as relatedness-supportive [46,48], as have the satisfying connections with others [46] and opportunity to collaborate [87] that might characterise group work. There were thus many opportunities for students in this study to realise their need for relatedness which, according to SDT, could have enhanced their motivation. MB,ChB students seemed to value the need for autonomy over the need for relatedness, as they spoke of finding the independent research work stimulating and rewarding. Nonetheless, many of them emphasised the importance of establishing a good relationship with a supervisor. Because many of these students had also had the experience of doing research in groups, however, they also recognised the benefits of working collaboratively, thereby satisfying their need for relatedness and in some cases enhancing their sense of competence through this collaborative work.

The findings show that simply having the opportunity to relate to others does not, in and of itself, enhance motivation. Relatedness was sometimes seen as coming at the expense of being able to optimise students’ own competence or performance, due to difficulties in delegating work and in resolving the conflict that seems inevitable in group work. This was frequently emphasised by allied health students, who were generally not given a choice about working in groups, although were largely free to choose their own group members. In this case, exercising this choice did not seem to counteract the potentially negative effects of group work-related challenges. Given the importance of peer acceptance and the acknowledgement of feelings and perspectives as relatedness-supportive factors [46], conflict in group work–or indeed, in supervisory relationships–could have the opposite effect on students’ need for relatedness, as well as their needs for autonomy and competence, and, therefore, their motivation. These students nonetheless still seemed to prioritise the need for relatedness over the need for autonomy, in part, perhaps because many of them felt that working in groups enhanced rather than constrained their performance (competence), in spite of the acknowledged difficulties of group work. It may be, then, that providing opportunities for relatedness can mediate the realisation–or lack thereof–of autonomy and competence.

These findings show that the provision of choice in the research curriculum should carefully consider how such opportunities may enable or restrict students’ abilities to realise their needs for autonomy and competence in particular, as these may sometimes counteract one another. The need for relatedness as it relates to motivation emerged less strongly in the data. This could be seen as a limitation of applying SDT in the context of this study. Nevertheless, we believe it provides insights into how relatedness-supportive factors in students’ learning environments could, in turn, facilitate the realisation of students’ autonomy and competence needs and thereby enhance their motivation to engage in a learning activity such as research.

## Limitations

There are limitations to this study. The focus on a South African higher education context and the small sample size may limit the transferability of the findings. However, using SDT as a theoretical lens has the potential more for analytical than for statistical transferability, allowing for general conclusions from a limited number of particular experiences to provide theoretical insights into other similar contexts [79]. There was under-representation from the Human Nutrition (no staff participants) and Physiotherapy (no student participants) divisions, which limits the conclusions that can be drawn regarding undergraduate research in these contexts. Similarly, this study did not include the perspectives of MB,ChB students who chose *not* to conduct research; including these perspectives in future studies would add a valuable dimension to understanding the intersection of choice, motivation, and research experiences. We recognise that students’ personal and sociocultural backgrounds, as well as their previous research and learning experiences and choice of degree programme, may affect their perceptions of undergraduate research. However, we believe that this is as much the case within programmes as across programmes and as such is not likely to account for observed variation across these two types of programmes. We further recognise that students’ perceptions around what research is might have influenced their responses and we did not explicitly explore this in the current study (see [66] for an exploration of this).

## Conclusion

The value of fostering undergraduate research skills is widely accepted. However, including research in medical and health sciences curricula poses several challenges. The element of student choice provides a useful dimension through which to view programmatic and institutional enablers and constraints of undergraduate research, using a Self-Determination Theory lens. This study has shown that the debate around choice and student motivation for engaging in research is still an unresolved one, particularly as the study highlighted that choice can function as both an enabler and a constraint on motivation. However, the study does provide insights for curriculum developers wishing to include a research component at undergraduate level. Specifically, while curriculum development tends to aim for a standardised, one-size-fits-all, this study highlights the importance of attending to individual-level factors when designing programmes with compulsory or elective research.

In order to optimise student engagement in research, careful scaffolding of research activities needs to be provided, allowing for sufficient choice to enable students to meet their needs for autonomy, competence, and relatedness. The provision for choice within research activities could be used to create conditions that are autonomy-supportive, competence-supportive, and relatedness-supportive, each of which may have variable, positive effects on motivation for different students. Regardless of whether research is compulsory or elective, providing choice within this activity–through choices about topics or supervisors, for example–could enhance an autonomy-supportive learning context. It is important to ensure that there is a balance between providing sufficient structure and enabling students to feel optimally challenged within that structure, in order to create competence-supportive conditions. Finally, allowing students choice with respect to their research supervisors or group members would allow for students for whom relatedness is an important component of motivation to optimise their engagement with the research task. This speaks to the need for flexibility and sensitivity to individual characteristics when designing undergraduate curricula.

## Supporting information

S1 Table Sample titles of undergraduate projects. (DOCX)

S1 Appendix Interview schedule. (DOC)

S2 Appendix Coding framework. (DOCX)
