# Supplementary material for: Perceived enablers and constraints of motivation to conduct undergraduate research in a Faculty of Medicine and Health Sciences: What role does choice play?
Source: PLoS One. 2019 Mar 13;14(3):e0212873. doi: 10.1371/journal.pone.0212873 (PMC6415790; doi:10.1371/journal.pone.0212873)
Supplement: S1 Table — (DOCX) [file pone.0212873.s001.docx]

S1 Table: Examples of undergraduate student project titles from data available 2013-2017

| **Degree programme** | **Quantitative studies** | **Qualitative studies** |
| --- | --- | --- |
| **Human Nutrition** | Assessment of risk of malnutrition in hospitalized children in Tygerberg Hospital, using the STAMP screening tool | N/A |
|  | An investigation into the internal and external factors contributing to food wastage in the Stellenbosch University residences, from the food provider’s point of view |  |
| **Occupational Therapy** | Establish concurrent validity between evidence-based assessments for fitness to drive and the driving simulator application | An exploration of play within a rural community |
|  | The relationship between bed height to leg length ratio as a possible risk factor to falls in older adults in residential care facilities | The factors influencing return to high school for learners with a spinal cord injury in the Western Cape: An adolescent perspective |
| **Physiotherapy** | The effectiveness of exercise therapy on pain and quality of life in office workers with non-specific neck pain compared to no therapeutic exercise | N/A |
|  | The effectiveness of dynamic exercise on muscle strength, functional ability and self-reported pain in patients with rheumatoid arthritis |  |
| **Speech-Language & Hearing Therapy** | A survey of the perceptions of a group of Grade 1 teachers in the Cape Town area regarding the use of oral narratives in teaching | The perceptions of Speech-Language Therapists at a tertiary hospital on communication with their clients: A qualitative case study |
|  | The knowledge and perceptions that prospective students have about a career in Speech-Language and Hearing Therapy. | Communication changes according to significant others of stroke survivors with little or no functional speech |
| **MB,ChB** | Testicular cancer in Tygerberg Hospital 2000-2015: A descriptive retrospective study | Intimate knowledge of a disease: experiences of medical students who develop TB |
|  | Determining student experience of clinical teaching in a South African academic hospital: Validating an adapted questionnaire | A tangled web of definitions: Deconstructing health science students’ concept of research |
