# Supplementary material for: Perceived enablers and constraints of motivation to conduct undergraduate research in a Faculty of Medicine and Health Sciences: What role does choice play?
Source: PLoS One. 2019 Mar 13;14(3):e0212873. doi: 10.1371/journal.pone.0212873 (PMC6415790; doi:10.1371/journal.pone.0212873)
Supplement: S1 Appendix — (DOC) [file pone.0212873.s002.doc]

**S1 Appendix Interview Schedule**

**Outline of proposed interview questions for student interviews**

*These questions will be used as a guide for the semi-structured, open-ended interviews with students.*

**What undergraduate degree were you or are you currently registered for in the FMHS?**

**Which department / division did you or are you conducting your research project in?**

**Please tell me about the research project that you conducted or are conducting as part of your undergraduate degree.**

(Prompt, for MBChB students: explore how the student got to their project: did they have an idea and subsequently approach the relevant department, or did they simply approach an “approachable” senior or did they approach a discipline that interests them, etc. Also explore when they started their project, and when they first started thinking of doing research).

(Prompt, for all students: what type of research it was (e.g. qualitative / quantitative) – and whether they would choose to do a different kind of research if they could do it again, and why).

**What have you found to be the most challenging aspects of doing this research?**

(Use prompts to unpack their responses further)

**What have you found to be the most rewarding aspects of doing this research?**

(Use prompts to unpack their responses further)

**Could you tell me about your experience of working in a group / individually on the research project? (How) do you think your experience would have been different had you worked alone / in a group?**

(Prompt: Explore preferences in this regard, and how working in one way or the other is perceived to either enhance or hinder the research process).

**Please tell me about your experience of obtaining ethics approval for your research.**

(Prompt: are there any ways in which you think this process could or should change in order to facilitate undergraduate research?)

(Prompt: how involved was your supervisor in assisting with the ethics application?)

**Do you think that your course/curriculum prepared you well enough to be able to undertake your research project?**

(Prompt: explore whether they did any research courses or modules as part of doing their research projects; whether they found these helpful and if not, why not. Explore what kind of content they think would be helpful in a research module that would assist them with their research project)

**What was your experience of supervision on your research project?**

(Prompt: how much input was given, whether enough, too much or too little; explore positives and/or negatives)

**Are there any ways in which you think you could have been better supported during your research – by your department, supervisor, and/or other support structures available to you in the FMHS?** (Prompt: are there any additional structures or mechanisms, as well as resources (e.g. resource materials, workshops) that you think would be helpful to students doing research in the FMHS?)

**Has your experience of research during your undergraduate degree influenced your choices regarding what you are doing or planning to do after completion of your undergraduate degree? Please elaborate.**

(Prompt: Based on your experience of doing research during your undergraduate degree, do you think you are likely to engage in research again in the future?)

**How would you advise a student with no experience who might be interested in undertaking a project?** (MBChB students) OR: **What advice would you have for students starting out on a research project?** (Allied Health Sciences students)

For current students only:

**Are you planning to publish your research?**

(If yes, explore their motivation for this and if they have a plan for doing so; if no, prompt reasons why)

**Have you had the opportunity to present your research?**

(If yes, explore when and where; if no, explore if they would have liked to, and if not, why not).

In addition to the above questions, the following are for past students / alumni only:

**For past students: How long ago did you graduate?**

**For past students: Have you been involved in research following the completion of your undergraduate research project? Please elaborate.**

**Outline of proposed interview questions for Faculty staff & support structure interviews**

*These questions will be used as a guide for the semi-structured, open-ended interviews with staff.*

Can you tell me about how you are involved in undergraduate research in the FMHS?

For other support structure staff: How does your division / unit contribute to enabling undergraduate research in the FMHS?

For department /divisional heads / research supervisors: What opportunities are there for students in your department / division for undergraduate students to conduct research?

**(NB: Probe** here specifically for how they structure opportunities for student research into their programme – e.g. for Allied Health Sciences, whether they have specific research modules, what these cover and when they are run, and whether students have structured time during year to do their research, have supervision etc.)

In your opinion, what are the main advantages of students conducting research during their undergraduate degrees? (Prompt: do you think that doing research in their undergraduate degrees is more or less likely to lead to them pursuing postgraduate study and or research during their careers?)

In your opinion, what are the primary challenges facing undergraduate students doing or wanting to do research as part of their undergraduate degrees?

Are there any ways in which you think the FMHS could better facilitate or support undergraduate research? Please elaborate.

Have you had direct involvement with an undergraduate research project, in the form of supervision? If yes, could you tell me about the challenges and rewards involved in providing this support?

On average, what percentage of the undergraduate research projects that you or your department have been involved in result in publications? And in conference presentations? How do you think this could be facilitated by the FMHS?

What is your perception of the current processes and requirements for ethical review of undergraduate research? (Prompt: are there any ways in which you think this process could or should change in order to facilitate undergraduate research?)

Do you think that funding is a major factor influencing students’ ability to conduct research? Please elaborate.

Are you aware of any funding sources that are targeted at supporting undergraduate research?
