# Supplementary material for: Perceived enablers and constraints of motivation to conduct undergraduate research in a Faculty of Medicine and Health Sciences: What role does choice play?
Source: PLoS One. 2019 Mar 13;14(3):e0212873. doi: 10.1371/journal.pone.0212873 (PMC6415790; doi:10.1371/journal.pone.0212873)
Supplement: S2 Appendix — (DOCX) [file pone.0212873.s003.docx]

**S2 Appendix Coding Framework**

**Coding framework: interviews**

**Curriculum**

(This theme covers the ways in which research is structured – or not structured – in the formal curriculum in various programmes, including the time that students have available to conduct research, the timing of how and when research takes place, types of research skills and training that students are equipped with a result of curriculum content, the extent to which students feel prepared for undertaking research, the degree of choice regarding research-related activities and requirements, and the amount of exposure (formal or informal) that students get to information about resources available to them for conducting their research)

- Ways in which research is placed in curriculum

(This theme speaks specifically to the ways in which research is structured into the curriculum – e.g. as part of a research module, an elective research component – as well as the content and structure of formal curriculum content / training relating to research knowledge and skills. This also includes the way in which the module/s or research activities are structured in terms of the scheduling or timing of when they occur in academic year)

- Time

(The ways in which participants speak about time as it relates to the curriculum or opportunities to conduct research – such as the protected time that is built into the curriculum for research activities to occur, and the timing of how and when research is conducted throughout the period)

- - *Protected / structured time*
- Research skills

(The research skills that students are equipped with as a result of this training or that are identified as being necessary or important for research)

- Preparedness for research / preparation

(Although linked to the above – research skills / training – this theme relates more to the participants’ subjective experiences of being prepared to conduct research, including more informal or extracurricular factors that they may list (e.g. prior degrees) in this regard)

- Choice within curriculum

(This theme speaks to the degree or choice that students have in relation to research activities, such as choice about their research topics, about whether participation in research is voluntary, and about the groups that they join to conduct the research)

- - *In terms of participation in research*
  - *In terms of nature / topic of research*
  - *In terms of groups*
  - *In terms of supervisors*
- Exposure to / knowledge of resources available

(The degree of formal (within curriculum) or informal (extracurricular) exposure that students get regarding resources that are available to them for conducting their research – such as information about funding support)

**Understandings of research**

(This theme relates to how students describe, perceive and / or understand ‘research’ as a concept and activity)

**Motivation**

(This theme relates to students’ motivations for conducting research, whether intrinsic or extrinsic)

- Intrinsic motivators for doing research

(This relates to intrinsic motivators for why students wanted to do research, including a sense of achievement or the desire to know more about a particular issue)

- Extrinsic motivators for doing research

(This relates to extrinsic motivators for why students embarked on research projects, including the incentive to obtain additional marks or the fact that it was compulsory in order to obtain their degree)

**Methodological preferences**

(This speaks to students’ perceptions of and preferences for particular methodological approaches – broadly, qualitative and quantitative research methods – and how this may have influenced their perceptions and understandings of the nature of research itself, as well as their enjoyment of the activity)

- Qualitative
- Quantitative

**Supervision**

- Supervisor roles & responsibilities (what they do or should do)

(Perceptions and understandings of what research supervisors’ roles and responsibilities are with respect to the research process – e.g. to provide input on the protocol, to assist with administrative processes such as ethics applications, etc.)

- Students’ roles & responsibilities – potentially collapse this into the supervisor code above and differentiate between student and supervisor within that code

(Perceptions and understandings of what students’ roles and responsibilities are in relation to the research / supervision process. This will also include differentiating specific roles for the research team, where students conduct research in groups)

- Supervisor involvement (how they are (subjectively) experienced in terms of their input)

(Experiences of the degree of involvement research supervisors had through each phase of the research process, including supervisor availability, accessibility, support, and feedback)

- Supervisor knowledge & experience (what they bring)

(Perceptions of the nature and scope of research supervisors’ knowledge and experience with respect to research)

- - *Clinical / content knowledge*
  - *Research knowledge*

**Funding**

(Broad theme that may be further broken down. Speaks to issues relating to funding research activities)

- Funding adequacy (perceptions of adequacy of current funding)
- Funding necessity (perceptions of the degree to which funding is necessary to enable research)
- Funding uses and influence on research outputs (perceived practical uses for funding or influence of funding on research outputs)
- Funding sources (awareness / lack of awareness of current funding sources)

**Ethics review**

(Broad theme that may be further broken down. Speaks to issues relating to the ethics application process)

- Time
- Administrative process
- Other permissions and applications

**Experience of doing research / fieldwork**

(What students have experienced during the research – e.g. liked sharing participant experiences, experienced resistance from participants, found certain other aspects rewarding or frustrating).

- Group work (the experiences students describe resulting from doing research as part of a group or, conversely, on their own. This distinguishes group experiences from simply the “choice” they have in choosing groups, which is coded under Curriculum as “Choice”)

**Outcomes of research**

(Tangible and intangible outcomes of the research process)

- Outputs / dissemination of research

(Experiences of and issues relating to presenting and publishing the findings of research)

- - *Presenting*
  - *Publishing*
- Personal outcomes

(How participants describe the personal outcomes / consequences of having conducted research. This may be linked to or include the code above on experiences of doing research)

- - *Influence on future research / lifelong learning*
  - *Confidence*
  - *Knowledge & skills*
